# High level expression of A2ARs is required for the enhancing function, but not for the inhibiting function, of γδ T cells in the autoimmune responses of EAU

**DOI:** 10.1371/journal.pone.0199601

**Published:** 2018-06-21

**Authors:** Dongchun Liang, Hui Shao, Willi K. Born, Rebecca L. O'Brien, Henry J. Kaplan, Deming Sun

**Affiliations:** 1 Doheny Eye Institute and Department of Ophthalmology, David Geffen School of Medicine at UCLA, Los Angeles, CA, United States of America; 2 Department of Ophthalmology and Visual Sciences, Kentucky Lions Eye Center, University of Louisville, Louisville, Kentucky, United States of America; 3 Department of Biomedical Research, National Jewish Health, Denver, CO, United States of America; Boston University School of Medicine, UNITED STATES

## Abstract

We previously reported that activated γδ T cells greatly enhance autoimmune responses, particularly the Th17 response. To determine the mechanisms involved, we made a series of comparisons between activated and non-activated γδ T cells. Our results showed that activated γδ T cells expressed greatly increased levels of A2A adenosine receptor (A2AR) and decreased amounts of CD73, as well as increased amounts of T cell activation markers such as CD69, CD44 and CD25. We show that A2AR is a major functional molecule in the enhancing activity of γδ T cells. A2AR^-/-^ γδ T cells (isolated from A2AR^-/-^ mouse), lost their Th17-enhancing activity as did A2AR^+/+^ γδ T cells (isolated from wt-B6 mouse) after treatment with an A2AR antagonist. Since γδ T cells possess either an enhancing or an inhibiting effect, we also tested whether A2AR expression on γδ T cells is essential to their inhibiting effect. Our results showed that the inhibiting effect of A2AR^-/-^ γδ T cells was as potent as that of A2AR^+/+^ γδ T cells. In a previous report we showed that the expression of different levels of CD73 molecule allowed γδ T cells to adjust their suppressive activity; in the current study, we show that expression of increased amounts of A2AR allows γδ T cells to more effectively exert their enhancing function.

## Introduction

γδ T cells can either enhance or inhibit immune responses [1,2], yet the mechanisms by which they do so are unclear. Clarification of these mechanisms should provide a better guide for therapeutic interventions. We previously demonstrated that the enhancing and inhibiting functions of γδ T cells are convertible and that the activation status of the γδ T cell determined the outcome [3–5]. The enhancing activity is elevated among activated γδ T cells, whereas the suppressive function dominates in non-activated γδ T cells [3–7]. A large portion (>60%) of the γδ T cells became activated and were a strong driving force on disease progression [3,4,8] during the pre-clinical phases (one week before the clinical appearance of disease) of induced mouse autoimmune uveitis (EAU). We have been searching for contributing molecules in an effort to determine whether the enhancing and inhibiting functions of γδ T cells are associated with the expression of specific surface molecules and to determine the underlying mechanism by which γδ cells switch their regulatory function. Our results showed that in addition to expressing increased amounts of T cell activation markers such as CD69, CD44 and CD25, activated γδ T cells also expressed greatly increased levels of A2A adenosine receptor (A2AR) and decreased amounts of CD73 [5,9]. Both molecules are crucially involved in metabolism, function, and the regulatory effect of extracellular ATP [10–12].

In a previous report, we showed that CD73 molecules play an important role in inhibiting the effect of γδ T cells [5]. CD73 converts AMP to adenosine, the expression of decreased amounts of CD73 molecules by activated γδ T cells results in a decreased ATP conversion to adenosine [5]. In the current study, we show that the expression of a high density of A2ARs favors the enhancing effect of γδ T cells, since the binding of increasing amounts of adenosine to γδ T cells diminishes adenosine binding by αβ T cells and dendritic cells (DC). Moreover, A2AR signaling promotes γδ T cell activation, whereas adenosine has an inhibiting effect on αβ T cells [9].

A2AR is a high-affinity adenosine receptor that is predominantly expressed on T cells [13–15]. Activation of A2AR suppressed the function of many immune cells such αβ T cells [11,16–19] and macrophage/DCs [14,17,18,20–27]. We previously reported that adenosine enhanced the responses of γδ and Th17 autoreactive T cell responses, while it inhibited Th1 responses [9]. A better understanding of how adenosine inhibits some immune responses but enhances others would be significant.

To further determine whether increased A2AR expression accounts for the augmented enhancing activity of activated γδ T cells, we compared the regulatory effect of A2AR^+/+^ and A2AR^-/-^ γδ T cells and assessed A2AR^+/+^ γδ T cell function, before and after treatment with an A2AR antagonist. Our results showed that γδ T cells lost most, if not all, of their enhancing activity and were less likely to be activated when A2ARs were functionally disabled. In contrast, the inhibiting function was retained. We conclude that a blockade of A2AR on γδ T cells could effectively regulate γδ activation, tipping the balance of the enhancing and inhibiting functions of γδ T cell, and could conceivably become a supplemental therapy for damping augmented autoimmune responses.

## Materials and methods

### Animals and reagents

All animal studies conformed to the Association for Research in Vision and Ophthalmology statement on the use of animals in Ophthalmic and Vision Research. Institutional approval (Protocol number: ARC#2014-029-03A) was obtained from the Institutional Animal Care and Use Committee of the Doheny Eye Institute, University of California Los Angeles, and institutional guidelines regarding animal experimentation were followed. Veterinary care was provided by IACUC faculty. Immunized animal that displays swelling joints were either be humanely euthanatized or administered an analgesic (buprenorphine, 0.1 mg/kg sc. twice daily or ketoprofen, 2 mg/kg sc. daily) until the swelling resolves. By the end of the study, mice were euthanized by cervical dislocation after an injection of over dosed Ketamine and xylazine prior to tissue collection.

Female C57BL/6 (B6) and TCR-δ^-/-^ mice on the B6 background, were purchased from Jackson Laboratory (Bar Harbor, ME). *A2AR*^-/-^ mice were kindly provided by Dr. Jiang-Fen Chen of Boston University [28]. Animals were housed and maintained in the animal facilities of the University of California, Los Angeles (UCLA). Recombinant murine IL-1, IL-7, and IL-23 were purchased from R & D (Minneapolis, MN). FITC-, PE-, or allophycocyanin-conjugated Abs against mouse CD73 (TY/11.8), CD44 (IM7), αβ T cell receptor (TCR, H57-597), or γδ TCR (GL3) and their isotype control Abs were purchased from Biolegend (San Diego, CA). PE-conjugated anti-mouse A2AR monoclonal Ab (7F6-G5-A2) was purchased from Santa Cruz Biotechnology (Dallas, TX). The non-selective AR agonist 50-N-ethylcarboxamidoadenosine (NECA), selective A2AR agonist 2-p-(2-carboxyethyl) phenethylamino-5’-N-ethylcarboxamidoadenosine (CGS21680), and selective A2AR antagonist (SCH 58261) [29,30] were purchased from Sigma-Aldrich (St. Louis, MO).

### Immunization procedure

B6 mice were immunized subcutaneously over 6 spots at the tail base and on the flank with 200 μl of emulsion containing 150 μg of the uveitogenic peptide IRBP_1-20_ [amino acids 1–20 of human interphotoreceptor retinoid-binding protein (IRBP; Sigma, St. Louis, MO)] emulsified in complete Freund’s adjuvant (CFA; Difco, Detroit, MI). Concurrently, 200 ng of pertussis toxin (PTX) (Sigma, St. Louis, MO) was injected intraperitoneally. At day 13 post-immunization, T cells were isolated from lymph node cells and spleen cells, then 1 x 10^7^ cells in 2 ml of RPMI 1640 medium (Cellgro, VA, USA) containing 10% fetal calf serum were added to each well of a 6-well plate (Costar) and stimulated for 48 h with 10 μg/ml of IRBP_1-20_ in the presence of 1 x 10^7^ irradiated syngeneic spleen cells as antigen-presenting cells (APCs) in the presence of either IL-12 (Th1 polarized) or IL-23 (Th17-polarized) (10 ng/ml), then activated T cell blasts were separated by Ficoll gradient centrifugation and cultured for another 72 h in the same medium used for stimulation without the peptide.

### T cell preparations

αβ T cells were purified from B6 mice immunized with the human interphotoreceptor retinoid-binding protein (IRBP) peptide IRBP_1-20_, as described previously [4,7,8], while γδ T cells were purified from immunized and control (naïve) B6 or *A2AR*^-/-^ mice. Nylon wool-enriched splenic T cells from naïve or immunized mice were incubated sequentially for 10 min at 4°C with FITC-conjugated anti-mouse γδ TCR or αβ TCR Abs and 15 min at 4°C with anti-FITC Microbeads (Miltenyi Biotec GmbH, Bergisch Gladbach, Germany); the cells were then separated into bound and non-bound fractions on an autoMACS^TM^ separator column (Miltenyi Biotec GmbH). The purity of the isolated cells was >95%, as determined by flow cytometric analysis using PE-conjugated Abs against αβ or γδ T cells. Resting γδ T cells were prepared either by isolation from naïve mice or by incubating activated γδ T cells in cytokine-free medium for 5–7 d, at which time they show down-regulation of CD69 expression [3]. Highly activated γδ T cells were prepared by incubating resting γδ T cells for 2 d with Abs against the γδ TCR (GL3) and CD28 (both 2 μg/ml, both from Bio-Legend, San Diego, CA), or cytokine combination (IL-1,IL-7 and IL-23).

### Competitive adenosine binding assay

A two-chamber assay was performed to evaluate the competitive binding ability of γδ T cells. αβ or DCs enriched from immunized TCR-δ^-/-^ mice were seeded in 24-well cell culture plates at a density of 2×10^6^/ml in 500 μl of complete medium, while γδ T cells were seeded at a density of 5×10^5^/ml in a cell culture insertion in 100 μl of complete medium and put into the wells with competitors. Cells were incubated at 37°C for 1 h with H^3^-adenosine at final concentrations of 1,000 nM in triplicate, then cell-bound and free H^3^-adenosine were separated by harvesting the cells on a cell harvester (Perkin Elmer) and the cell-associated radioactivity was measured by liquid scintillation. Relative adenosine binding ability of γδ to αβ T cells or to splenic cells was represented by their relative radioactivity of CPM.

### CFSE assay

Purified αβ T cells from IRBP_1-20_-immunized B6 mice were stained with CFSE (Sigma-Aldrich) as described previously [31]. Briefly, the cells were washed and suspended as 5x 10^6^ cells/ml in serum-free RPMI 1640 medium (Corning Cellgro, Manassas, VA), then incubated at 37°C for 10 min with gentle shaking with a final concentration of 5 μM CFSE. The cells were washed twice with RPMI 1640 medium containing 10% fetal calf serum (Atlantic Inc. Santa Fe, CA) (complete medium), suspended in complete medium, stimulated with immunizing peptide in the presence of irradiated syngeneic spleen cells as antigen-presenting cells (APCs), and analyzed by flow cytometry at 5 d after stimulation.

### Cytokine assays

Purified αβ T cells (3x10^4^ cells/well; 200 μl) from the draining lymph nodes and spleens of IRBP_1-20_-immunized B6 mice were cultured in complete medium at 37° C for 48 h in 96-well microtiter plates with irradiated syngeneic spleen APCs (1x10^5^) in the presence of 10 μg/ml of IRBP_1-20;_ a fraction of the culture supernatant was then assayed for IL-17 and IFN-γ using ELISA kits (R & D).

### Statistical analysis

The results in the figures are representative of one experiment, which was repeated 3–5 times. The statistical significance of differences between groups in a single experiment was initially analyzed by ANOVA; if statistical significance was detected, the Student–Newman–Keuls post-hoc test was subsequently used. P values less than 0.05 were considered a statistically significant difference and marked with one *; when P<0.01, two ** were used.

## Results

### A2AR^-/-^ γδ T cells have limited enhancing effect on autoimmune responses

**Fig 1:**
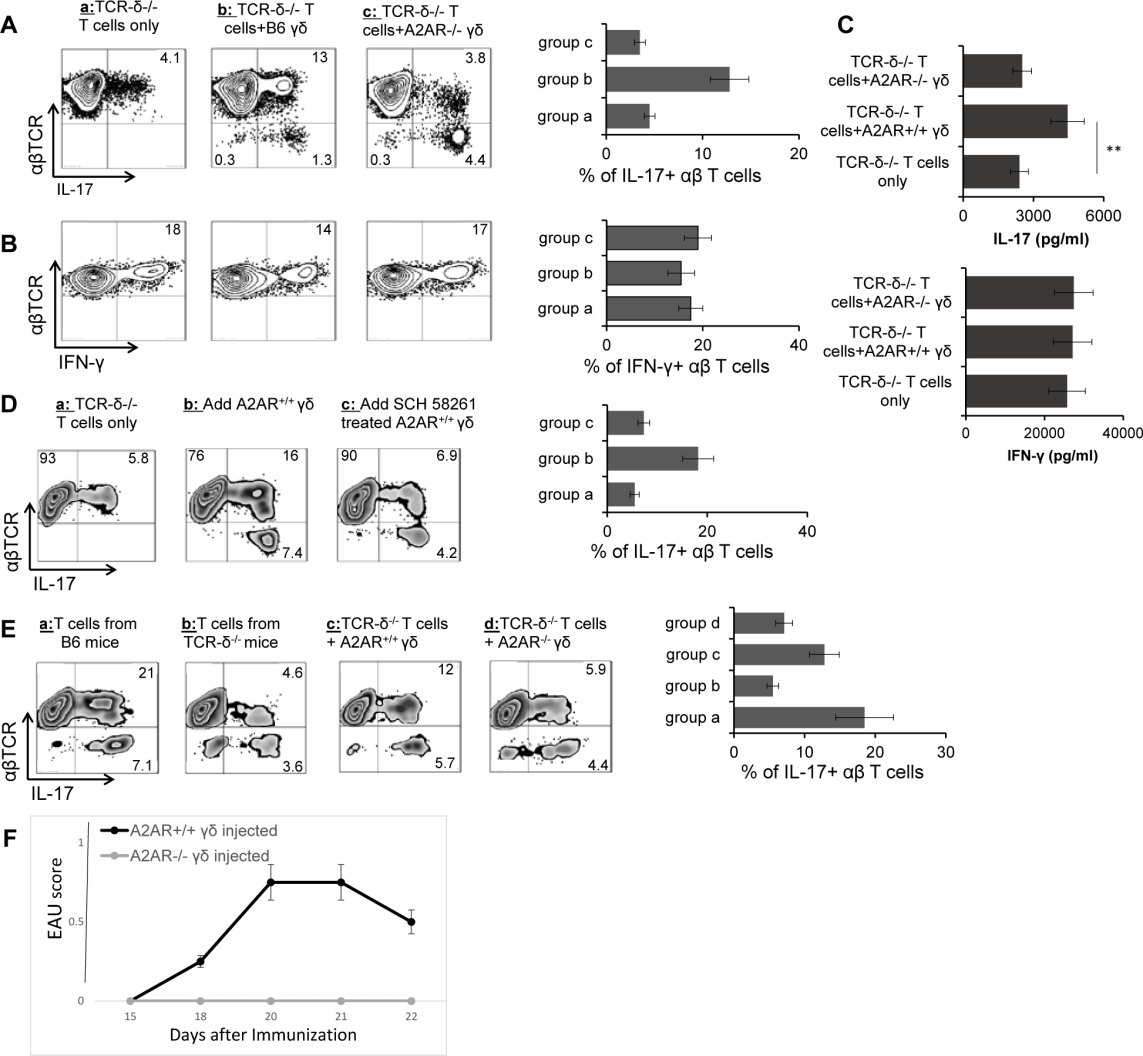
A2AR^-/-^ γδ T cells have a limited enhancing effect on autoimmune responses. A &B) A2AR^+/+^ enhanced the Th17 response, but A2AR^-/-^ γδ T cells did not; and neither enhanced the Th1 response. A2AR^+/+^ and A2AR^-/-^ γδ T cells were isolated from immunized wt-B6 or A2AR^-/-^ mice, respectively. Responder αβ T cells (1 x 10^6^/well) prepared from IRBP_1-20_ immunized TCR-δ^-/-^ mice on day 13 post-immunization were stimulated for 5 days with immunizing antigen and APCs under Th17 (A) of Th1 (B) polarized conditions with or without addition of 2% (2 x 10^4^/well) A2AR^+/+^or A2AR^-/-^ γδ T cells. The numbers of αβTCR^+^ IL-17^+^ and αβTCR^+^ IFN-γ^+^ cells were calculated by FACS analysis after a 5-day in vitro stimulation. C) Addition of A2AR^+/+^ γδ T cells, but not of A2AR^-/-^ γδ T cells, to αβ responder T cells enhanced IL-17 production but did not affect IFN-γ production. Production of IL-17 and IFN-γ by immunized TCR-δ^-/-^ mice was assessed by ELISA, after in vitro stimulation with the immunizing peptide (IRBP_1-20_), in the presence of A2AR^+/+^ or A2AR^-/-^ γδ T cells (2%). Cytokine amounts were evaluated in the culture cell supernatants by ELISA after a 48 h stimulation. D) Treatment of A2AR antagonist abolished the enhancing effect of A2AR^+/+^ γδ T cells. A2AR^+/+^ γδ T cells were added to responder αβ T cells in culture, with or without a pre-incubation with SCH58261, a selective A2AR antagonist (100nM) [29,30]. Results show that blockade of A2AR ligation abolished the enhancing effect of A2AR^+/+^ γδ T cells. E) Administration of TCR-δ^-/-^ mice with A2AR^+/+^γδ T cells, but not with A2AR^-/-^ γδ T cells, enhanced the Th17 response. Groups (n = 4) of TCR-δ^-/-^ mice were injected with A2AR^+/+^ or A2AR^-/-^ (1 x 10^6^/mouse) γδ T cells, before they were immunized with a pathogenic dose of IRBP_1-20_/CFA. At 13 days post-immunization, the Th17 responses were assessed 5 days after in vitro stimulation. The results shown are representative of those from five experiments. Differences were considered significant when *P* ≤ 0.05 (asterisk) and very significant when *P* ≤ 0.01 (two asterisks). F.) TCR-δ^-/-^ mice injected with A2AR^+/+^, but not A2AR^-/-^, γδ T cells were more susceptible to induced EAU. Groups of TCR-δ^-/-^ mice were immunized with IRBP _1-20_/CFA with or without a prior injection of 1 x 10^5^ (per mouse) A2AR^+/+^ or A2AR^-/-^ γδ T cells. EAU was scored as previously reported [3,4,8]. The results of two separate experiments are shown.

Our previous studies have established reliable assays for testing the effect of γδ T cells on autoimmune responses, in vitro and in vivo [3,4,32–35]. Testing of the adenosine effect used responder αβ T cells prepared from immunized TCR-δ^-/-^ mice because the αβ T cell response to adenosine is strongly affected by <1% of γδ T cells [3,4,32–35]. T cell responses to the immunizing antigen were determined in the absence or presence of γδ T cells [3,4,32–35]. As expected, the addition of activated γδ T cells, but not of non-activated, γδ T cells, significantly enhanced the Th17 but did not enhance the Th1 response [5,7,8]. We have compared the enhancing effect on autoreactive T cell responses of A2AR^+/+^ and A2AR^-/-^ γδ T cells, isolated from immunized wt-B6 or A2AR^-/-^ mice, respectively. The αβ T cell responses were assessed under either Th1- (addition of IL-12 and anti-IL-4 antibodies during in vitro stimulation, Fig 1B) or Th17-polarized conditions (Fig 1A, addition of IL-23 during in vitro stimulation) by monitoring intracellular expression of IFN-γ or IL-17 of the responder T cells [7,8]. As shown in Fig 1A, the number of IL-17^+^ T cells among the responder T cells was significantly increased if A2AR^+/+^ γδ T cells were added, but was not enhanced if the A2AR^-/-^ cells were added. ELISA tests measuring cytokine production of the responder T cells (Fig 1C) supported the prediction that A2ARs are crucially involved in γδ T cells’ enhancing function; whereas the Th1 response or production of IFN-γ showed no significant difference when either type of γδ T cells was added.

We then determined whether treatment of A2AR^+/+^ γδ T cells with an A2AR antagonist (SCH 58261) would affect their enhancing effect (Fig 1D). A2AR^+/+^ γδ T cells were added to responder αβ T cells, with or without pre-incubation with an A2AR antagonist. The results showed that pre-treatment of A2AR^+/+^ γδ T cells with an A2AR specific antagonist significantly decreased the enhancing effect.

We previously reported that injection of a small number (< 1 x 10^6^/mouse) of γδ T cells to TCR-δ^-/-^ mice significantly enhanced the autoreactive T cell responses of the recipients [3,4,32]. To assess the in vivo effect of A2AR^+/+^ or A2AR^-/-^ γδ T cells (Fig 1E), the TCR-δ^-/-^ mice were injected with either A2AR^+/+^ or A2AR^-/-^ γδ T cells (1 x 10^6^/mouse) before immunization, and the cytokine levels in the serum as well as the autoreactive T cell responses of the recipients were examined 13 d post-immunization (the time point at which the greatest T cell changes are seen) [4,32,36]. As shown, the number of IL-17^+^ IRBP-specific αβ T cells of TCR-δ^-/-^ recipients of A2AR^+/+^ γδ T cells increased 4 fold as compared to non-injected mice and 2–3 fold as compared to the TCR-δ^-/-^ recipients of A2AR^-/-^ γδ T cells. Serum cytokine levels agreed with the prediction that IL-17 levels were significantly increased in recipients of A2AR^+/+^γδ T cells, but not in recipients of A2AR^-/-^γδ T cells (Not shown). Adoptive transfer studies (Fig 1F) showed that IRBP-specific T cells isolated from TCR-δ^-/-^ mice injected with A2AR^+/+^, but not those injected with A2AR^-/-^, γδ T cells were more susceptible to EAU, suggesting that A2AR is an important molecules on γδ T cells for their enhancing activity.

#### A2AR agonist showed an increased inhibitory effect in the absence of γδ T cells

**Fig 2:**
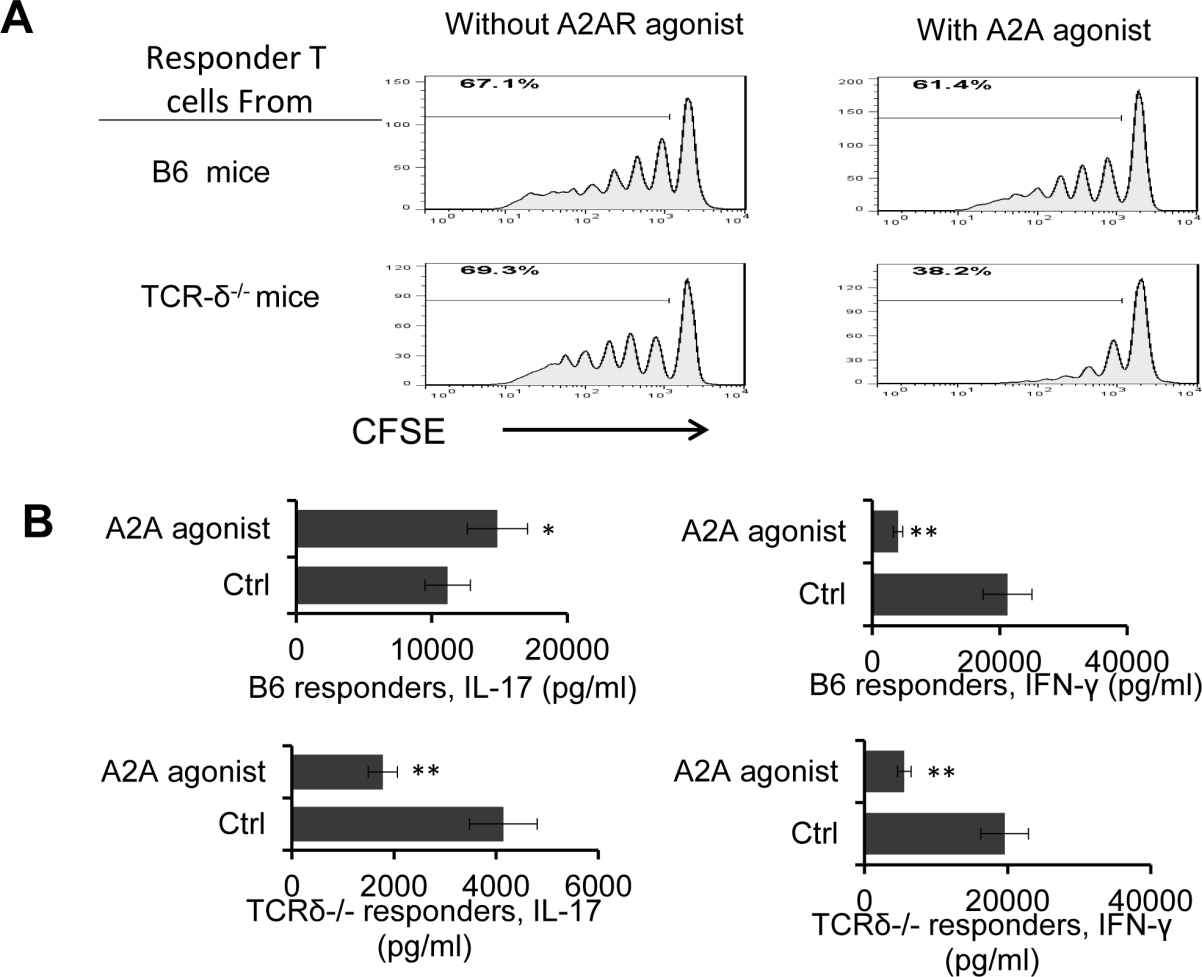
A2AR agonist is more inhibitory in the absence of γδ T cells. A) A2AR agonist inhibited TCR-δ^-/-^ responder T cells more effectively than it inhibited the B6 responder T cells. The αβ responder T cells (1 x 10^6^/well) were isolated from IRBP_1-20_-immunized B6 (upper panels) or TCR-δ^-/-^ mice (lower panels). The CFSE-labeled αβ T cells were stimulated for 5 d with immunizing peptide and APCs in the absence or presence of A2AR agonist. Cell proliferation was assessed by FACS analysis. B) In vitro treatment of A2AR agonist enhanced the Th17 responses when added to B6 responder T cells but inhibited the Th17 responses when added to TCR-δ^-/-^ responder T cells. Comparison of IL-17 and IFN-γ production by immunized αβ T cells isolated from immunized B6 or TCR-δ^-/-^ mice after in vitro stimulation, in the absence or presence of A2AR agonist, showing that A2AR agonist inhibited the Th1 response but enhanced the Th17 response of B6 cells, but not of TCR-δ^-/-^ T cells.

One way to determine the effect of γδ T cells in T cell responses is to compare T cell responses between wt-B6 and TCR-δ^-/-^ mice. Th1 CD3^+^ responder T cells prepared from immunized wt-B6, but not those prepared from immunized TCR-δ^-/-^ mice, consistently contain approximately 5% γδ T cells [3,7]. The results demonstrated in Fig 2A showed that A2AR agonist (CGS21680, 250nM) more effectively inhibited the response of TCR-δ^-/-^ than it did that of B6 T cells. Cytokine tests showed that in the absence of γδ T cells (T cells of TCR-δ^-/-^ mice) (Fig 2B, lower panel) the αβ T cell response was inhibited; but in the presence of γδ T cell (B6 T cells), the inhibitory effect of the A2A agonist on Th17 response is reversed, suggesting that the γδ T cells among the B6 responder T cells offset the suppressive effect of adenosine, leading to a higher Th17 response. Comparison of the A2AR agonist effect on IFN-γ production demonstrated, however, that the A2AR agonist inhibited the IFN-γ responses regardless of the appearance of γδ T cells (Fig 2B, right panels).

#### γδ T cells enhance the autoreactive T cell response by competing with the binding of adenosine

**Fig 3:**
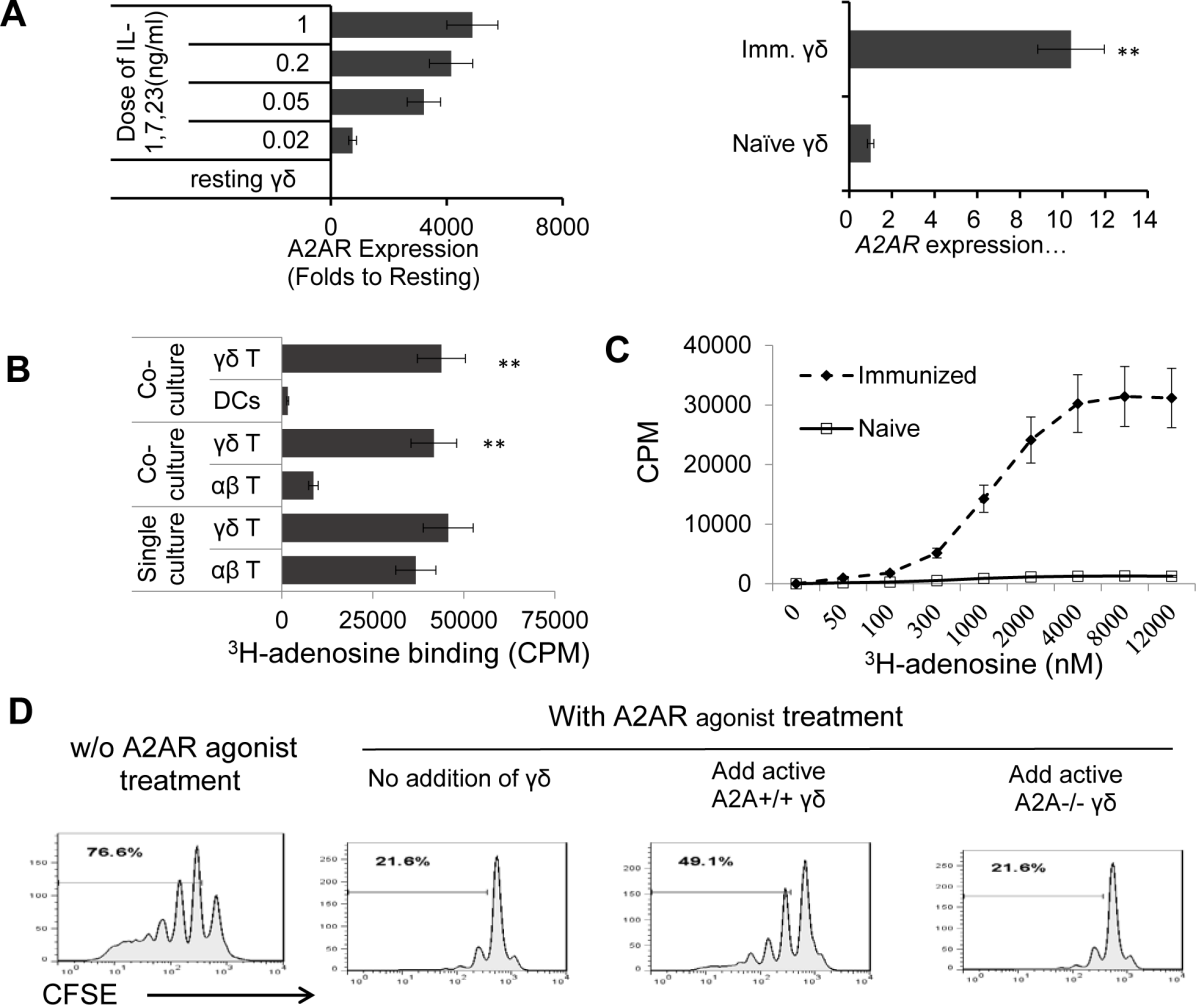
γδ T cells enhance the autoreactive T cell response by competing with the binding of adenosine. A) Activated γδ T cells express an increase of >1000-times the A2AR but only minimally increased A2BR. Activated γδ T cells and naïve γδ T cells were isolated from immunized from naïve B6 mice, respectively. The expression of A2AR/A2BR was determined by real-time PCR assay. Real-time RT-PCR analysis of A2AR and A2B transcripts among total RNA isolated from αβ and γδ T cells isolated from naïve and IRBP_1-20_-immunized B6 mice before and after in vitro activation. qPCR was performed with Gapdh as the internal reference. Results were represented as 2^-ΔCt^. **, p < 0.01. B) Competitive binding test revealed a hierarchical order of adenosine capture by immune cells as γδ T cells >> αβ T cells >> DCs. αβ responder T cells were isolated from IRBP_1-20_-immunized TCR-δ^-/-^ mice. DCs were from 5 d GM-CSF-cultured bone marrow cells. In a 24-well plate the T cells were seeded at 1 x 10^6^/well and DCs at 5 x 10^5^/well. After incubation with1,000 nM H^3^-labeled adenosine for 1 h, the cells were harvested, and cell-bound H^3^-adenosine was counted by liquid scintillation. ** p < 0.01. C) Comparison of adenosine binding of γδ T cells isolated from naïve and immunized mice γδ T cells from immunized or naïve B6 mice, respectively. 1 x 10^5^/well cells were cultured in 96-well plate for 48h. 0–12,000 nM H^3^-labeled adenosine for 1 h, then the cells were harvested and cell-bound H^3^-adenosine counted by liquid scintillation. D) A2AR^+/+^γδ T cells, but not A2AR^-/-^ γδ T cells, possess an enhancing effect, which converted the inhibitory effect to enhancing on the αβ T cell response of an A2AR agonist. αβ and γδ T cells (1 x 10^5^) were isolated from IRBP_1-20_-immunized TCR-δ^-/-^ mice. The responder αβ T cells were labeled with CFSE and incubated for 5 d with immunizing peptide in the presence of APCs alone (A) or in the presence of the A2AR^+/+^ or A2AR^-/-^ γδ T cells (B), in the absence or presence of an A2AR agonist, and with or without an addition of A2AR^+/+^γδ T cells. The results shown are representative of three experiments.

We previously reported that γδ T cells expressed significantly greater numbers of A2ARs as compared to αβ T cells and DCs [9]. We wanted to determine whether increased absorption of the AR agonist by γδ T cells via A2ARs accounted for the enhancing effect. Real-time PCR study showed that cytokine activated γδ T cells express an increase of >1000-times the A2AR (Fig 3A, left panels), whereas they expressed only an increase of 10-times the A2BR as compared to naïve γδ T cells (Fig 3A, right panels).

To further test whether increased adenosine-binding activity of γδ T cells accounts for the observed enhancing effect by removal of adenosine binding to responder αβ T cells, we performed a competitive adenosine-binding test using a radioactively labeled adenosine. For this assay, the γδ T cells were co-cultured, separated by chamber separators, with αβ T cells or macrophages/DCs. H^3^-labeled adenosine was added to the cultures 6 h before cell harvest and the incorporated amounts of the isotope on γδ, αβ T cells or macrophages/DCs were assessed by a β counter. Binding tests (Fig 3B) revealed that the hierarchical order of adenosine capture by immune cells was γδ T cells >> αβ T cells >> DCs. The αβ or γδ T cells could bind comparable amounts of adenosine if cultured alone; however, the γδ T cells have a much greater adenosine-binding effect than DCs or αβ T cells if they are co-cultured in separated two-chambers, suggesting that the binding of H^3^-labeled adenosine by αβ T cells and DCs dramatically declined in the presence of activated γδ T cells, and that γδ T cells enhance the autoreactive T cell response by competing with the binding of adenosine. We previously reported that activation confers the increased adenosine-binding activity to γδ T cells [9]. Fig 3C shows that γδ T cells isolated from immunized mice, but not those isolated from naïve mice, expressed a greatly increased adenosine binding activity.

**Fig 4:**
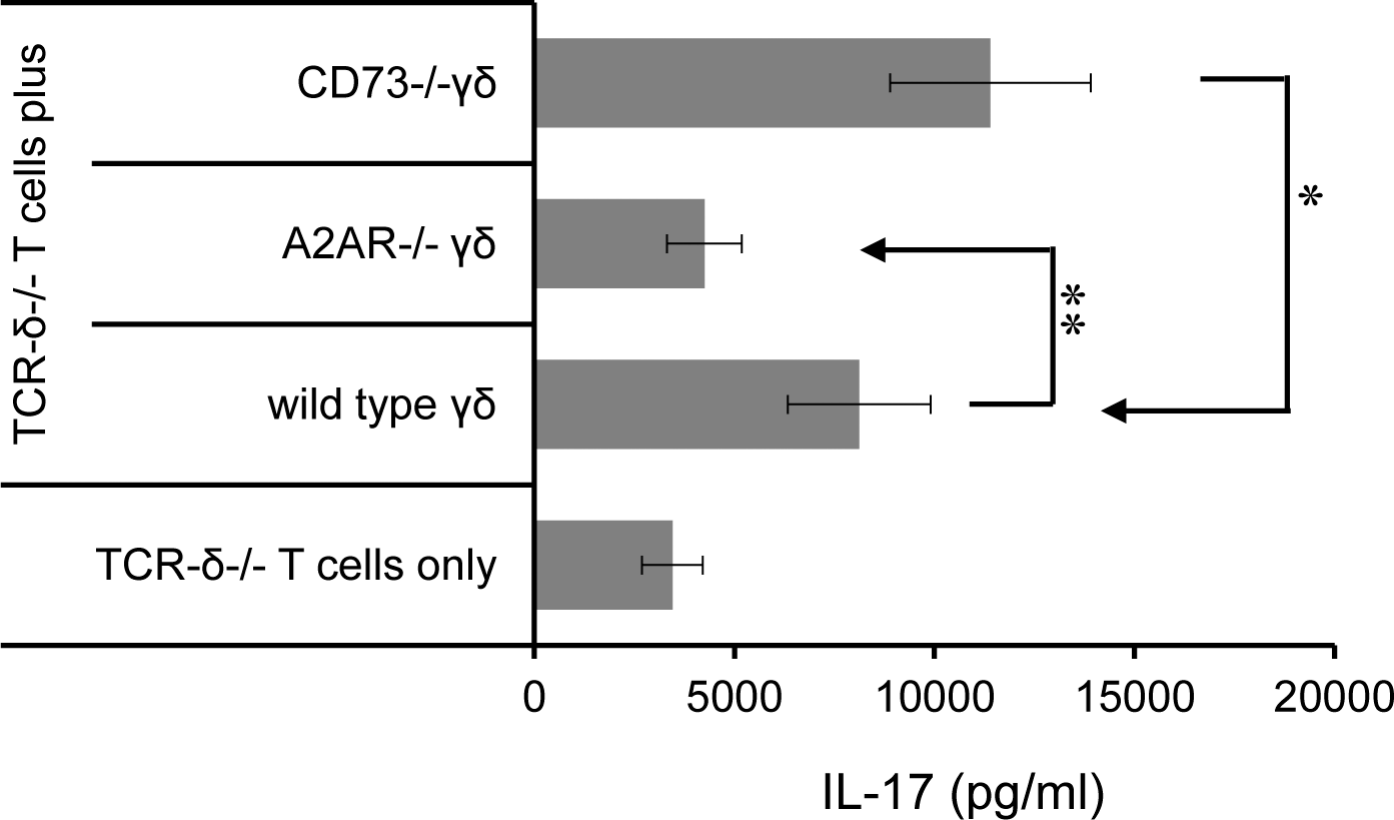
CD73^-/-^ γδ T cells retained the enhancing activity. The enhancing effect of A2AR^+/+^, A2AR^-/-^, and CD73^-/-^ γδ T cells were compared for promoting cytokine (IL-17) production of responder T cells obtained form TCR-δ^-/-^ mice. Responder T cells were stimulated with the immunizing antigen and APCs in the absence or presence of indicated γδ T cells. The 48h cultured cell supernatants were sampled for testing IL-17 production by ELISA.

To prove the prediction that expression of A2AR is functionally essential for γδ T cells, responder αβ T cells isolated from immunized TCR-δ^-/-^ mice were CFSE-labeled and stimulated with the immunizing peptide and APCs with or without added A2AR^+/+^ or A2AR^-/-^ γδ T cells, in the absence or presence of A2AR agonist. The proliferation of CFSE-labeled αβ T cell was assessed by FACS analysis 5 d after stimulation (Fig 3D). The results shown in Fig 3D demonstrated that the addition of A2AR agonist inhibited the proliferation of αβ responders in the absence of γδ T cells and that its inhibitory effect is weakened in the presence of A2AR^+/+^ γδ T cells, but not in the presence of A2AR^-/-^ γδ T cells. The results suggest that γδ T cells enhance the autoreactive T cell response by competitively binding adenosine via A2AR. We have compared the enhancing effect of A2AR^+/+^, A2AR^-/-^, and CD73^-/-^ γδ T cells in promoting cytokine (IL-17) production (Fig 4), our results showed that CD73^-/-^ γδ T cells retained the enhancing activity, implying that altered expression of A2AR, but not CD73, by activated γδ T cells are responsible.

#### Failure to express A2AR did not affect γδ T cells’ suppressive activity

Since γδ T cells can either enhance or inhibit an immune response [5,6,37–39], we wished to determine whether γδ T cells with disabled A2AR function have altered suppressive function. Our previous studies determined that the inhibitory effect of γδ T cells was enhanced when exogenous AMP was provided and that increased degradation of AMP to adenosine by the CD73 molecules on γδ T cells accounted for the effect [5,10,40]. Inhibiting tests were done using a CFSE assay, in which the responder αβ T cells, isolated from immunized TCR-δ^-/-^ mice, were pre-labeled with CFSE and stimulated with the immunizing peptide and APCs (Fig 4). In the presence of AMP, both A2AR^+/+^ and A2AR^-/-^ γδ T cells showed a comparable effectiveness in suppression, whereas both cells showed a significantly diminished suppressive activity if tested in the absence of AMP. Importantly, the A2AR^+/+^ and A2AR^-/-^ γδ T cells demonstrated a comparable suppressive effect.

#### A2AR dysfunction compromised γδ T cells’ activation

**Fig 5:**
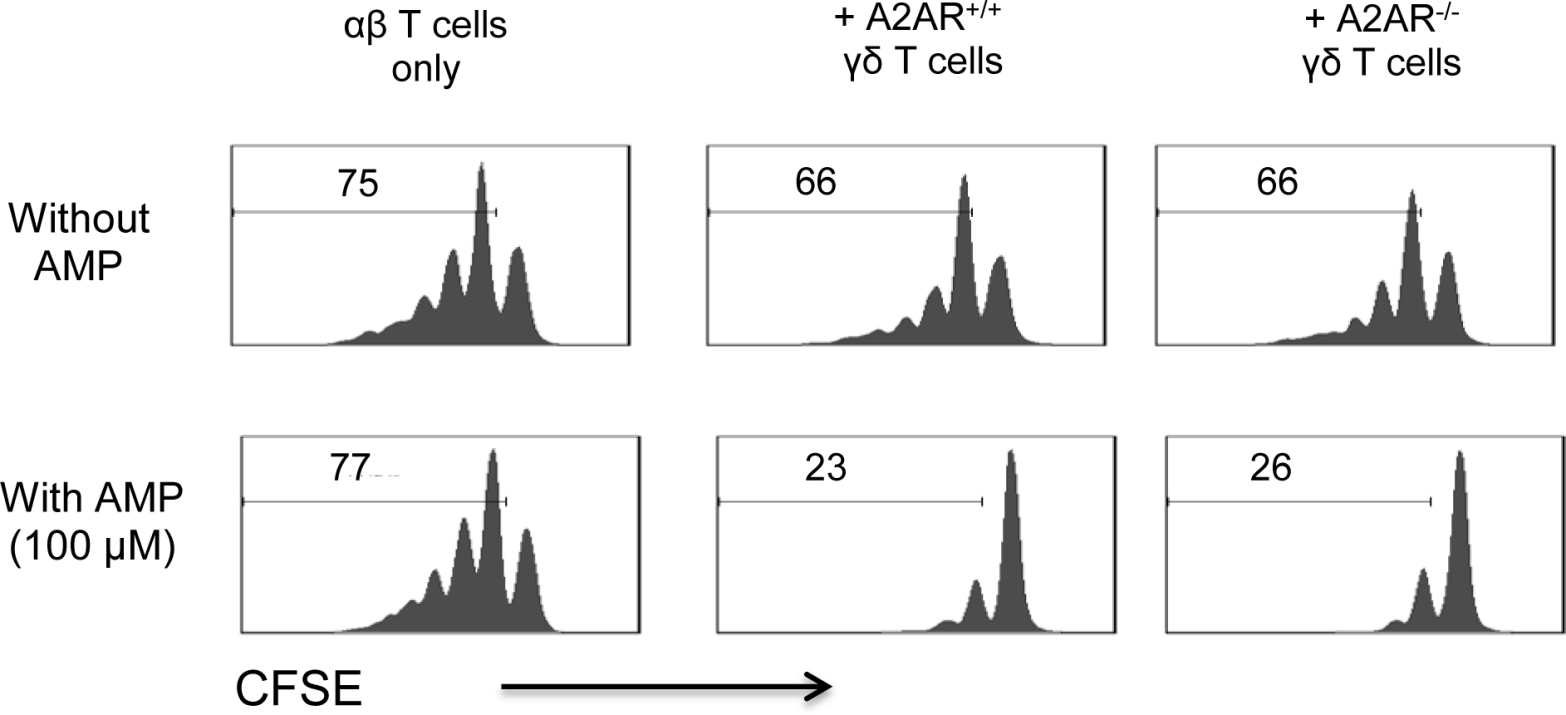
The suppressive effects of A2AR^-/-^ and A2AR^+/+^ γδ T cells are not distinguishable. Proliferative responses of CFSE-labeled αβ T cells isolated from immunized TCR-δ^-/-^ B6 mice (1 x 10^5^/well) were assessed after in vitro incubation for 1 h with immunizing peptide and APCs alone (upper panels) or in the presence of 10 μM AMP (lower panels), 5% A2AR^+/+^ γδ T cells (Middle panels), or 5% A2AR^-/-^ γδ T cells (right panels). The results shown are representative of those obtained in three separate experiments.

**Fig 6:**
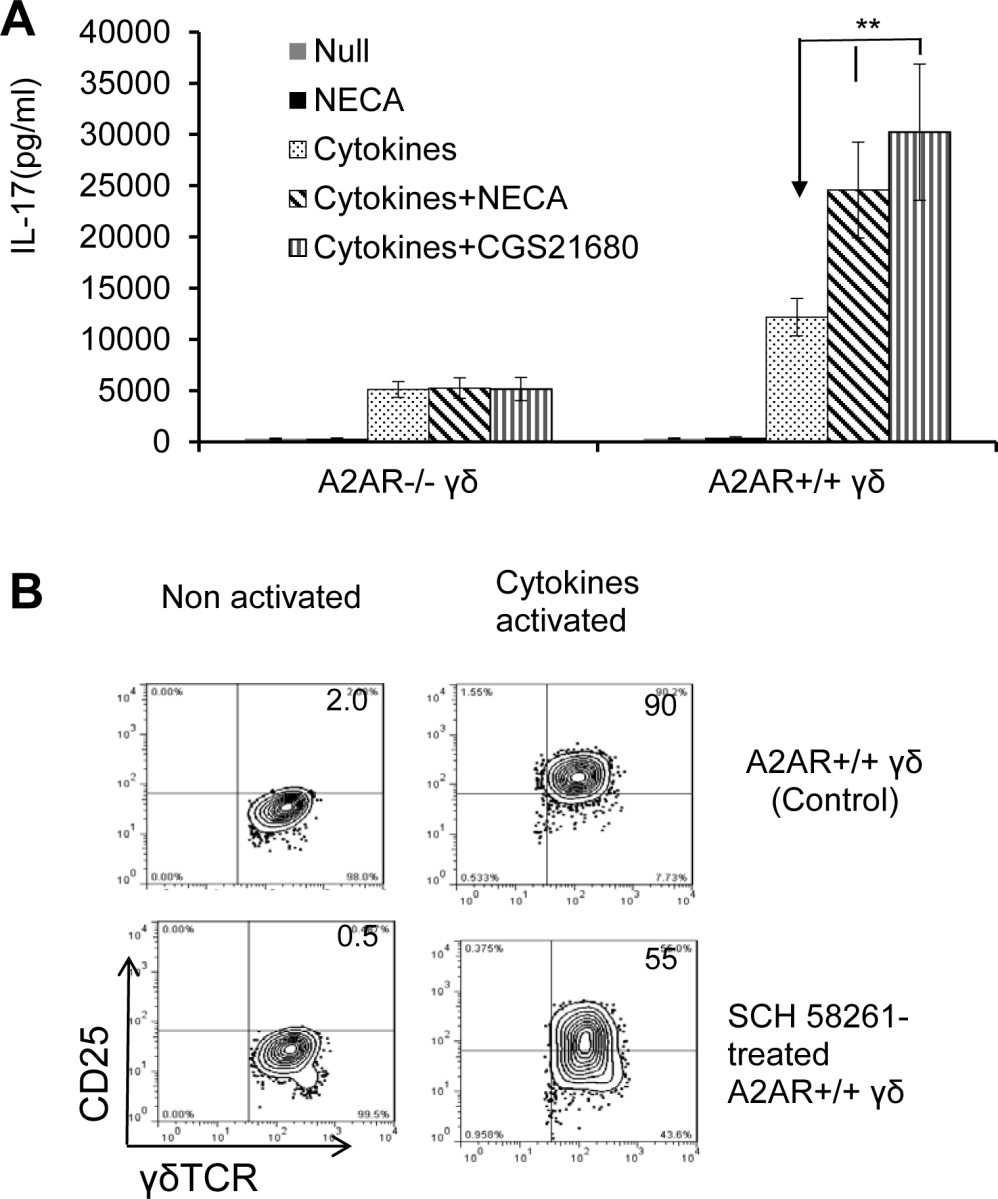
A2AR dysfunction compromised γδ T cells’ activation threshold. A) Failure to express A2AR rendered γδ T cells less activated. In a 24-well plate, 2 x 10^5^ A2AR^+/+^ or A2AR^-/-^ γδ T cells were incubated with medium only (Control), with pooled cytokines (IL-1, IL-7, and IL-23), and with or without or NECA (300 nM) or the A2AR-specific agonist CGS21680. At 48 h after the culture, IL-17 amounts in the culture supernatants were assessed by ELISA. B) Blockade of A2AR expression prevented γδ T cell activation. Surface staining for the T cell activation marker CD25 showing that A2AR antagonist treated A2AR^+/+^ γδ T cells express low amounts of CD25 after exposure to cytokines. The results shown are from a single experiment and are representative of those obtained in >5 experiments. **_,_ p < 0.01.

Previous studies have shown that cytokines are potent stimulators of γδ T cell [37,38,41,42]. We were able to show that adenosine has an amplifying effect on the cytokine-mediated γδ activation [5,9]. To determine whether dysfunction of A2AR affects γδ activation, we exposed A2AR^+/+^ and A2AR^-/-^ γδ T cells to a determined amount of pooled cytokines (IL-1+IL7+IL-23) [5,9], with or without the addition of an adenosine analogue NECA (300 ng/ml). Fig 5 shows that NECA and the A2AR-specific agonist CGS21680 significantly amplified A2AR^+/+^ γδ T cells’ IL-17 production induced by cytokine Mixture. No such synergistic effect could be found in the cytokine response of A2AR^-/-^ γδ T cells (Fig 6A), which reacted less to cytokines; and the addition of A2AR agonist showed no enhancing effect, further supporting the notion that A2AR on γδ T cells crucially regulates the activation of γδ T cells and thus the enhancing effect of γδ T cells. We also compared the activating response of γδ T cells, before and after A2AR antagonist treatment. The wt-γδ T cells treated with A2AR antagonist expressed significantly decreased amounts of activation markers when activated by cytokines (Fig 5B, upper panels). However, when A2ARs were blocked by an A2AR antagonist, cytokine-induced CD25 expression on γδ T cells was decreased, indicating that A2ARs on γδ T cells are crucially involved in γδ T cell activation. Fig 6B shows the result of CD25 staining. The same results were observed on the expression of additional T cell activation markers such as CD44 and CD69 (data not shown).

## Discussion

Studies in mouse models of autoimmune diseases (EAU & EAE) have demonstrated that the development of autoimmune diseases is closely associated with increased activation of γδ T cells [7,43]. It was hypothesized that activated γδ T cells exacerbate the autoimmune development [3–5]; however, the mechanism remains to be clarified. In previous reports we have demonstrated that activated γδ T cells exacerbate EAU development by enhancing Th17 autoimmune responses. Our studies have repeatedly shown that this enhancing effect was due to activated γδ T cells. In addition, we have revealed that the γδ T cells’ inhibiting and enhancing functions are both strongly affected by extracellular ATP metabolism [44]. To define structural and functional change between activated and non-activated γδ T cells in correlation to their functional switch, our previous studies demonstrated that activated γδ T cells expressed elevated amounts of A2AR but decreased amounts of CD73 molecules [5,9]. Both molecules are crucially involved in metabolism, function, and the regulatory effect of extracellular ATP [10–12].

Adenosine is an endogenous purine nucleoside that diffuses to surrounding cells, where it binds to specific cell surface receptors after its release from cells and after being formed by the breakdown of nucleotides. Early studies have shown that extracellular adenosine accumulation protects cells and tissues from injury caused by an augmented inflammatory response and immune-mediated damage [12] [18,27,45–48]. Supporting data include that lymphocytes from A2AR^-/-^ mice show higher rates of cell proliferation and produce high amounts of IFN-γ levels upon stimulation [49]. Engagement of A2AR inhibits IL-12 production but increases IL-10 production by human monocytes [50,51]. In vivo treatment with an A2A agonist decreases intestinal inflammation [48]. Treatment of T lymphocytes with adenosine or the A2AR agonist inhibits proliferation [21,52] and inhibits the secretion of cytokines, including IL-2, TNF-α, and IFN-γ [21,53]. Nevertheless, aberrant adenosine signaling has been implicated in various pathological conditions [11,18,54].

Since most previous studies examined the adenosine effect on Th1-type immune responses, we sought to determine whether Th17-type immune responses are similarly affected. To our surprise, adenosine had many different effects on αβ and γδ T cells [5,9,35], and it differs greatly with respect to its activation of Th1 and Th17 autoimmune responses. Moreover, unlike its suppressive effect on αβ T cells and DCs [27,48,55], adenosine enhanced the γδ T cell response and Th17 autoreactive T cell responses [9]. We have found that adenosine is an important factor in γδ T cell activation; that γδ T cells participate in the conversion of extracellular ATP to adenosine [5,9]; and that γδ T cells bind adenosine much more strongly compared to immune cells such as αβ T cells and DCs so that these cells were less affected by adenosine in the presence of γδ T cell [9,35]. Further study on the issues of how ATP/adenosine metabolites and γδ T cells regulate each other’s function should improve our understanding of autoimmune pathogenesis and our understanding of how γδ T cells exert their regulation effects, which will lead to better treatment of the disease.

In this study we determined whether increased expression and increased adenosine binding ability of A2AR on activated γδ T cells is accountable for their enhancing or inhibiting function. The availability of A2AR^-/-^ mice [28] gave us the opportunity to test whether the A2AR^+/+^ and A2AR^-/-^ γδ T cells were functionally distinct in their enhancing and inhibiting effects. We also tested whether blockade of A2ARs on A2AR^+/+^ γδ T cells using a specific A2AR antagonist affected their enhancing or inhibiting function. The results of both approaches led to the conclusion that A2ARs on γδ T cells are critical regulatory molecules; in the absence of functional A2AR, the enhancing effect of γδ T cells was mostly abolished.

We have shown that expression of a high density of A2AR is essential for activated γδ T cells to exert their enhancing effect; however, the inhibitory effect of the γδ T cells is not affected by this molecule. As a consequence, the net effect of enhancing and inhibiting was balanced toward the former. Our results demonstrated therefore that blockade of A2AR might be a successful strategy for damping augmented autoimmune responses.

Our results demonstrated that an augmented enhancement of autoimmune responses by γδ T cells relies on expression of higher levels of A2ARs. Overexpression of the high-affinity A2ARs enables γδ T cells to effectively remove excess adenosine, which would otherwise bind to αβ T cells and DCs. Given that adenosine is a co-stimulating molecule in γδ activation [9], elevated A2AR on γδ T cells would decrease the activation threshold of γδ T cells and augment γδ T cell activation, leading to enhanced autoimmune responses.

In a previous report, we demonstrated that CD73 molecules expressed on γδ T cells are functionally important for γδ T cells’ suppressive function [5]. As an effective enzyme that converts AMP to adenosine, the expression of decreased amounts of CD73 molecules by activated γδ T cells results in a decreased ATP conversion to adenosine [5], consequently leading to augmented immune responses.

In summary, Purinergic signaling plays a central role in inflammation [10,56–58]. The incomplete understanding of varied roles of the different purinergic signaling events in physiological and pathophysiological processes has been hindering the application. In a previous study we demonstrated that expression of downregulated CD73 allowed activated γδ T cells to exhibit downgraded suppression and thus shift the enhancement and inhibition toward the latter. In the current study, we show that A2ARs are important molecules that are required for γδ T cells to exert their enhancing activation. Blockade of A2AR function on γδ T cells should allow us to tip the enhancing and inhibiting function of γδ T cells toward the latter.

## Supporting information

S1 File The ARRIVE guidelines checklist. (PDF)
