# Correction: High level expression of A2ARs is required for the enhancing function, but not for the inhibiting function, of γδ T cells in the autoimmune responses of EAU

**DOI:** 10.1371/journal.pone.0207546

**Published:** 2018-11-08

**Authors:** Dongchun Liang, Hui Shao, Willi K. Born, Rebecca L. O'Brien, Henry J. Kaplan, Deming Sun

The following information is missing from the Funding section: This work was supported by Research to Prevent Blindness, New York, New York.
